# Effects of genome-wide copy number variation on expression in mammalian cells

**DOI:** 10.1186/1471-2164-12-562

**Published:** 2011-11-16

**Authors:** Richard T Wang, Sangtae Ahn, Christopher C Park, Arshad H Khan, Kenneth Lange, Desmond J Smith

**Affiliations:** 1 Department of Molecular and Medical Pharmacology, David Geffen School of Medicine, University of California, Los Angeles, CA 90095, USA; 2 Department of Electrical Engineering, Signal and Image Processing Institute, School of Engineering, University of Southern California, Los Angeles, CA 90089, USA; 3 Department of Biostatistics, School of Public Health, University of California, Los Angeles, CA 90095, USA; 4 Department of Human Genetics, David Geffen School of Medicine, University of California, Los Angeles, CA 90095, USA; 5 GE Global Research Center, One Research Circle KW-C1308, Niskayuna, NY 12309, USA

## Abstract

**Background:**

There is only a limited understanding of the relation between copy number and expression for mammalian genes. We fine mapped *cis *and *trans *regulatory loci due to copy number change for essentially all genes using a human-hamster radiation hybrid (RH) panel. These loci are called copy number expression quantitative trait loci (ceQTLs).

**Results:**

Unexpected findings from a previous study of a mouse-hamster RH panel were replicated. These findings included decreased expression as a result of increased copy number for 30% of genes and an attenuated relationship between expression and copy number on the X chromosome suggesting an *Xist *independent form of dosage compensation. In a separate glioblastoma dataset, we found conservation of genes in which dosage was negatively correlated with gene expression. These genes were enriched in signaling and receptor activities. The observation of attenuated X-linked gene expression in response to increased gene number was also replicated in the glioblastoma dataset. Of 523 gene deserts of size > 600 kb in the human RH panel, 325 contained *trans *ceQTLs with -log_10 _*P *> 4.1. Recently discovered genes, ultra conserved regions, noncoding RNAs and microRNAs explained only a small fraction of the results, suggesting a substantial portion of gene deserts harbor as yet unidentified functional elements.

**Conclusion:**

Radiation hybrids are a useful tool for high resolution mapping of *cis *and *trans *loci capable of affecting gene expression due to copy number change. Analysis of two independent radiation hybrid panels show agreement in their findings and may serve as a discovery source for novel regulatory loci in noncoding regions of the genome.

## Background

Radiation hybrid (RH) panels were originally devised to build high resolution maps of mammalian genomes [1,2]. The panels are created by lethally irradiating a donor cell (mouse, human, rat, etc) harboring a selectable marker and propagating the resulting DNA fragments by fusing the donor cells with the recipient hamster cell line A23. Each clone in an RH panel contains a random assortment of the donor DNA permitting construction of a physical map. Since high doses of radiation can be used, a large number of breakpoints can be obtained, > 10^4 ^in a typical panel of ~100 clones.

RH panels exhibit copy number variation (CNV) for essentially all genes and represent a powerful resource for unbiased examination of CNV-induced effects on gene expression. The existence of CNVs across multiple clones in a panel boosts statistical power.

Studies that map quantitative trait loci (QTLs) regulating gene expression (expression QTLs or eQTLs) usually rely on naturally occurring polymorphisms as a source of genetic variation and meiotic recombination to narrow down the regulatory loci. Frequently, the mechanistic significance of naturally occurring polymorphisms in affecting gene expression is not immediately apparent from the context. Genetic alterations due to CNVs have recently come to the fore as a source of considerable polymorphism in humans [3,4]. In contrast to other polymorphisms, a one to one correspondence between copy number and gene expression is, on its face, a reasonable expectation for CNVs although exceptions have been noted [5-7]. Since the variation in RH cells is due to CNVs, we refer to loci affecting expression in the RH panels copy number eQTLs or ceQTLs. However, unlike naturally occurring CNVs, variation in the RH panels is uniform and genome-wide.

Recently, array comparative genomic hybridization (aCGH) and gene expression microarrays were used to fine map loci regulating expression genome-wide in a mouse-hamster radiation hybrid panel [8]. The analysis of the mouse RH panel revealed a number of unexpected findings. These included the fact that ~30% of genes showed decreased gene expression in response to increased copy number, a potentially novel form of dosage compensation for the × chromosome independent of × chromosome inactivation, and the existence of ceQTLs in noncoding regions of the genome.

To further investigate these surprising findings, we used the Stanford G3 radiation hybrid panel [9]. The 83 clones in this panel are derived from a human male donor genome. We also used publicly available glioblastoma multiforme (GBM) data from The Cancer Genome Atlas (TCGA). We found consistent overlap between the human RH, mouse RH and TCGA data in terms of regulated pathways for genes with negative correlation between CNVs and expression data and attenuated response of X-linked genes in response to copy number increase. In addition, we found ceQTLs in non-genic regions in the two RH datasets and that these nongenic ceQTLs could not be explained by recently discovered exotic transcripts in noncoding regions harboring ceQTLs.

## Results

### Gene expression

RNA was extracted from each of the 79 available radiation hybrid clones and technical replicates hybridized to Illumina HumanRef-8 v1.0 BeadChips. The relative hybridization efficiencies of hamster and human transcripts on the arrays were comparable (Additional File 1 Figure S1A-D) and there was good reproducibility between duplicate arrays (Additional File 2 Figure S2).

### Assessing copy number and retention frequency in the G3 RH panel

To measure DNA copy number in the RH cell lines, we used array comparative genomic hybridization (aCGH) of each clone compared to the reference hamster A23 recipient line. The aCGH genotyping agreed well with the historical PCR genotyping (χ^2 ^= 159,996, 1 d.f., *P *< 2.2 × 10^-16^) (Additional File 3 Figure S3A). The average loss of PCR markers across all RH cells was 36.6%. This loss is likely due to the multiple passages of the RH clones since its creation a decade ago. Almost no gain of markers (< 1%) was observed. Individual cell lines showed large variation in loss, ranging from 3-96%. The final retention frequency (i.e., average amount of donor DNA retained per RH clone) was 11.4%. The average donor DNA fragment length was 4 Mb.

Across the 79 available clones in the G3 RH panel, the entire human genome is represented, on average, nine times (0.11 × 79 = 9) (Additional File 3 Figure S3B), although a few regions were extreme. As expected, the retention of the region surrounding thymidine kinase (*Tk1*), the selectable marker used in creating the panel was 100%. Human centromeric regions were preferentially retained in the RH cell lines (Welch's *t*-test, *P *< 10^-15^) (Additional File 3 Figure S3C-E), as found previously [9]. This observation implies that human centromeres function efficiently in hamster cells despite lineage differences [10].

### Gene expression changes with copy number

**Figure 1:**
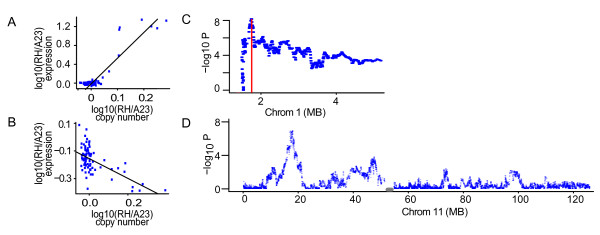
***Cis *and *trans *human ceQTLs**. (A) *Trans *ceQTL. *RPL18P4 *on chromosome 7 displays an increase in log_10_(RH/A23) gene expression as log_10_(RH/A23) copy number of a marker at 53.7 Mb on chromosome 19 increases (α = 5.2, -log_10 _*P *= 8.35). (B) Example of negative α *trans *ceQTL. Log_10_(RH/A23) gene expression of *DPH3 *on chromosome 3 decreases as log_10_(RH/A23) copy number of a marker at 128.1 Mb on chromosome 12 increases (α = -0.72, -log_10 _*P *= 4.27). (C) *Cis *ceQTL for *GNB1 *is located at 1.7 Mb on the beginning portion of chromosome 1. Red line denotes position of regulated gene. (D) Multiple *trans *ceQTLs on Chromosome 11 regulating *RPS13 *on chromosome 1. The peaks located at 17.1 Mb and 47 Mb show strongest evidence of regulation. Points are aCGH markers plotted along chromosomes. Centromeres are grey.

We used a previously described linear regression model [8] to relate changes in gene expression to copy number in the 79 available clones (Methods). Briefly, the log_10 _(RH expression/A23 control) of each gene served as the dependent variable while the log_10 _(RH/A23) CGH intensity served as the regressor. The change in expression due to copy number is characterized by an effect size parameter α (Figure 1A, B). If α = 1, gene expression is exactly proportional to copy number. The significance of α was assessed by permutation testing (Methods).

**Figure 2:**
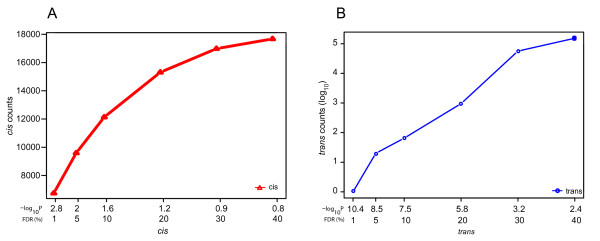
***Cis *and *trans *RH ceQTLs at varying FDR thresholds**. Counts of loci regulating gene expression as a result of increased copy number in *cis *(A) and *trans *(B). FDR and corresponding -log_10 _*P *are shown. Note that *trans *counts are log_10 _scale.

We defined a *cis *ceQTL as a locus within a 5 Mb radius of a regulated gene (Figure 1C). *Trans *ceQTLs were defined as loci regulating genes at a distance greater than 5 Mb or on another chromosome (Figure 1D). To account for multiple hypothesis testing, we applied false discovery rates (FDRs) to *cis *and *trans *ceQTLs separately [11,12]. We used an FDR threshold of < 0.25 for our ceQTLs in the human data giving 15,263 *cis *ceQTLs. In mouse, the same threshold gave 16,234 *cis *ceQTLs. For *trans *ceQTLs, we also used FDR < 0.25 in both mouse and human data. These FDRs correspond to *P *values of 0.09 and 2.92 × 10^-5 ^for *cis *and *trans *ceQTLs, respectively in the human RH data (Figure 2A-B).

**Figure 3:**
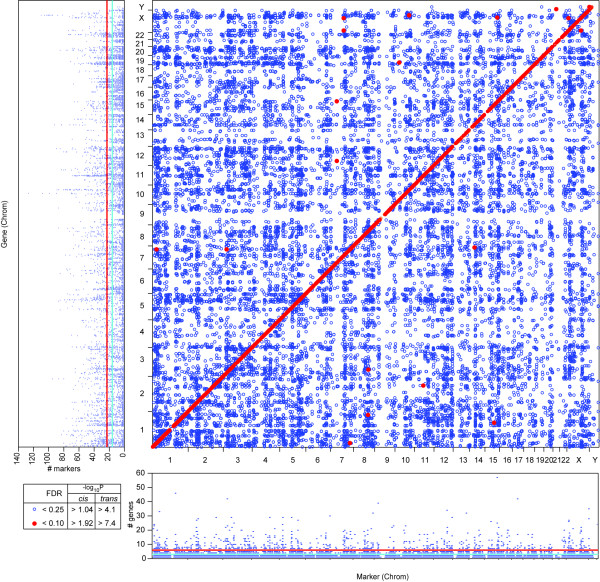
***Cis *and *trans *ceQTLs**. Every point represents the regulation of a gene (y-axis) by a marker (x-axis). White streaks are centromeric regions. Horizontal marginal shows number of genes regulated in *trans *by each loci. The probability that a marker would regulate more than 4 genes is 0.31 (FDR < 0.4, light blue line) and more than 7 genes is 0.008 (FDR < 0.01, red line). Vertical marginal illustrates number of *trans *ceQTLs regulating each gene. The likelihood that a gene is regulated by more than 16 trans ceQTLs is 0.29 (FDR < 0.3, light blue line) and more than 23 is 0.01 (FDR < 0.01, red line). Both highly regulatory loci and highly regulated genes are evident.

Figure 3 shows the landscape of ceQTLs at various FDRs. Consistent with other eQTL mapping studies [13], we found *trans *bands which may indicate hotspots of regulatory activity (Figure 3 horizontal marginal). Genes regulated by multiple loci (Figure 3 vertical marginal) may represent key genes integrating multiple pathways. The high breakpoint density of the RH panel permitted multiple regulatory loci along individual chromosomes to be resolved (Figure 1D).

### *Cis *ceQTLs and genes that turn down their own expression

The median distance between a gene and its *cis *ceQTL was 531 kb (Additional File 4 Figure S4A). *Cis *ceQTL effect sizes (α) showed a bimodal distribution with means of 0.73 and -0.12 for positive and negative α's, respectively. (Additional File 4 Figure S4B). A total of 5,831 of 16,234 (36%) *cis *ceQTLs decreased their gene expression when their copy number was increased (i.e., possessed negative α). In our mouse RH, 30% of *cis *α were negative at FDR < 0.25.

After identifying ~11,000 orthologous genes between mouse and human, we determined the number of common genes whose expression correlated with copy number to be 7,936. Human and mouse possessed 6,092 and 5,979 genes whose expression increased with copy number respectively and 1,844 and 1,957 genes whose expression was inversely correlated with copy number respectively. 4,805 genes had positive α and 670 had negative α in both human and mouse data. A chi-square test showed enrichment of both positive *cis *α and negative *cis *α ceQTLs across both species (*P *< 2.2 × 10^-16^), suggesting that negative *cis *α ceQTLs are not simply due to noise. Using a more stringent cutoff of FDR 5% in the human RH data, 198 negative correlations still persist. In the mouse RH data at an FDR of 5%, 172 negative correlations still exist and the overlap of 32 genes with negative α is statistically significant (P = 1.04 × 10^-7^).

We employed DAVID [14] to search the Gene Ontology for functional enrichment of genes with negative *cis *α in the human and mouse RH datasets (Table 1). There was a high degree of functional conservation for genes with negative *cis *α with the most enriched categories involving membrane functions, receptor activity and signaling. Consistent with this observation, a Mann-Whitney rank test of the GO scores between the two data sets was significant (P = 1.34 × 10^-7^). The trend was less clear among genes with positive *cis *α in the mouse and human (Additional file 5 Table S1) with categories such as metabolic process, regulation of apoptosis and binding highly conserved between the two species.

**Table 1:** GO Enrichment for negative cis α at FDR < 0.25

Human	Score	FDR	Mouse	Score	FDR
**Biological Process**					

System Development	5.55 × 10^-38^	8.77 × 10^-35^	System Development	2.22 × 10^-19^	3.45 × 10^-16^

Cell-Cell Signaling	1.57 × 10^-36^	2.49 × 10^-33^	Organ Development	1.08 × 10^-15^	1.73 × 10^-12^

Cell Differentiation	1.88 × 10^-24^	2.97 × 10^-21^	Cell Differentiation	4.86 × 10^-14^	7.57 × 10^-11^

Ion Transport	3.11 × 10^-24^	4.93 × 10^-21^	Cell-Cell Signaling	6.67 × 10^-12^	1.04 × 10^-8^

Organ Development	1.06 × 10^-23^	1.68 × 10^-20^	Positive Regulation of Biological Process	2.94 × 10^-10^	4.58 × 10^-7^

**Cellular Component**					

Plasma Membrane Part	2.38 × 10^-53^	3.53 × 10^-50^	Plasma Membrane	1.60 × 10^-27^	2.30 × 10^-24^

Plasma Membrane	3.62 × 10^-46^	5.37 × 10^-43^	Extracellular Region	1.04 × 10^-15^	1.43 × 10^-12^

Intrinsic To Plasma Membrane	5.56 × 10^-45^	8.26 × 10^-42^	Plasma Membrane Part	9.65 × 10^-14^	1.38 × 10^-10^

Integral To Plasma Membrane	9.54 × 10^-44^	1.42 × 10^-40^	Synapse Part	4.67 × 10^-12^	6.70 × 10^-9^

**Molecular Function**					

Passive Transmembrane Transporter Activity	2.87 × 10^-27^	3.75 × 10^-24^	Passive Transmembrane Transporter Activity	2.06 × 10^-11^	2.56 × 10^-8^

Substrate-Specific Transmembrane Transporter Activity	3.85 × 10^-18^	5.03 × 10^-15^	Substrate-Specific Transmembrane Transporter Activity	4.49 × 10^-7^	5.59 × 10^-4^

Receptor Binding	1.55 × 10^-13^	2.03 × 10^-10^	Receptor Binding	2.05 × 10^-5^	2.54 × 10^-2^

Receptor Activity	9.80 × 10^-13^	1.28 × 10^-9^	Heme Binding	1.07 × 10^-4^	1.3 × 10 ^-1^

A recent study of cells trisomic for each of the mouse chromosomes 1, 13, 16 and 19 [15] provided an opportunity to test our negative *cis *α ceQTLs and further rule out noise as a cause of this surprising phenomenon. Similar to the analysis of the RH panels, we used linear regression to estimate effect sizes due to copy number increases in the aneuploid cells. Out of 1,699 orthologous genes between mouse RH and mouse trisomy data, 1,275 and 1,191 *cis *ceQTLs had positive *cis *α in the trisomy and mouse RH data respectively and 424 and 508 possessed negative *cis *α respectively. A chi-square test showed enrichment of both positive *cis *α and negative *cis *α ceQTLs (*P *= 7.4 × 10^-9^). We repeated this test using human RH and mouse aneuploidy data (1,213 orthologous genes) and found a highly significant overlap of 131 genes with negative cis α (*P *= 8.2 × 10^-12^). The replicability of the negative α findings across these datasets argues in favor of a true biological phenomenon.

Absolute expression levels of genes with positive cis α is statistically significant from genes with negative cis α (P = 4.5 × 10^-9^), although this difference is due to a fraction of highly expressed genes with positive cis α (Additional File 6 Figure S5A). The mean gene expression values were quite close (12.04 versus 11.99, positive and negative α respectively). *Cis *ceQTLs with negative alpha show little evidence of antisense transcription (289 out of 5,831) and the genes underlying them were largely found in their entirety across all 79 RH cell lines (Additional File 6 Figure S5B). In addition, neighboring markers nearly always had concordant α (Additional File 6 Figure S5C-D).

### Decreased *cis *effects on X chromosome

**Figure 4:**
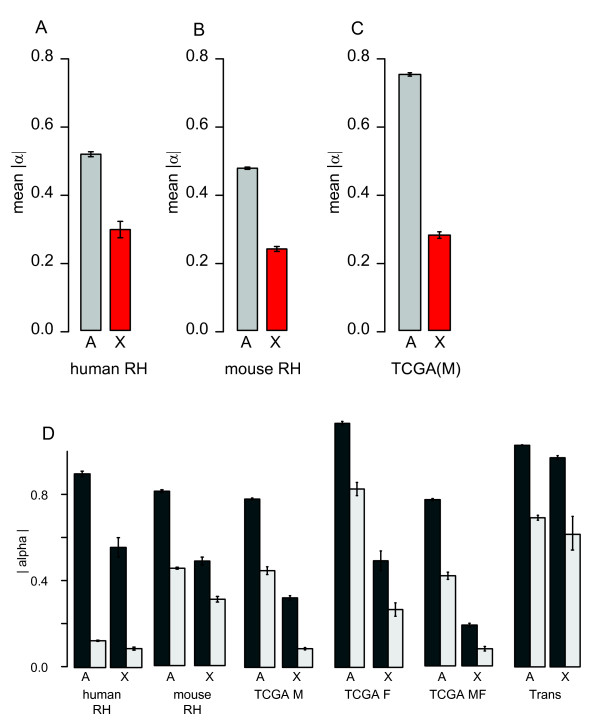
***Cis *ceQTLs on autosomes versus X chromosome**. Mean absolute value *cis *α on autosomes and X chromosomes for (A) human RH, (B) mouse RH and (C) TCGA male. (D) Mean absolute value *cis *α for positive (dark grey) and negative (light grey) ceQTLs on the autosomes and X chromosome in human RH, mouse RH, male TCGA, female TCGA and all TCGA. Mean absolute value α for X-linked genes regulated in *trans *are also shown. Error bars are s.e.m.

Genes on the X chromosome in the human RH dataset showed a significantly attenuated *cis *response to increased copy number compared to the autosomes (Figure 4A). The same phenomenon was also found in our mouse dataset, where the mean *cis *α on the X chromosome was significantly less (roughly half) than the autosomes [8] (Figure 4B). We performed a paired t-test by evaluating the average positive *cis *α for autosomes and X chromosomes for each human RH clone and found a significant difference (*P *= 6.7 × 10^-9^, 78 d.f.) between the two. The same was true for negative *cis *α (*P *= 1.9 X 10^-13^, 78 d.f.) (Figure 4D). Both the human donor and A23 hamster cells used to construct the hybrids are male. Because donor chromosomes are fragmented by irradiation and concatenated to other random fragments upon incorporation into the recipient cell line, possible activation of the donor *Xist *locus would not consistently silence all X chromosome genes. Since the retention frequency of the donor X chromosome is only 5.9% (Additional File 3 Figure S3F), activation of the recipient hamster *Xist *locus would render the RH clones functionally haploid for most of the X chromosome and would be inviable. Thus the attenuated *cis *ceQTL effect size on the X chromosome implies a *Xist*-independent dosage compensation mechanism for X-linked genes.

### *Cis *ceQTLs in cancer and RH cells have similar properties

Using glioblastoma multiforme (GBM) cancer data publicly available from the Cancer Genome Atlas (TCGA) project, we applied linear regression to estimate *cis *copy number effects on gene expression and then compared the results to our human and mouse RH panels. While not a perfect analogue to RH panels, cancer often possess alterations in copy number which would be expected to influence gene expression. In the cancer data, 38.7% of the human genome showed copy number variation. X chromosomal coverage was 68.8%. Similar to the RH analysis, we employed a 5 Mb radius for *cis *effects and corrected *P *values such that FDR < 0.05 (*P *< 0.04).

At FDR < 0.05, 8,764 genes in the TCGA dataset showed *cis *effects between copy number and gene expression of which 5,815 were common to the human RH data. Human RH and TCGA had 4,670 and 5,320 *cis *ceQTLs with positive α and 1,145 and 495 *cis *ceQTLs with negative α. Enrichment of *cis *ceQTLs that had positive and negative α in both data sets (4,372 and 197 for positive and negative α respectively) was demonstrated by chi-square (*P *< 2.2 × 10^-16^). The same was true for TCGA and mouse RH (*P *= 2.5 × 10^-11^). The number of genes with negative correlation between copy number and gene expression in common between the TCGA, human RH and mouse RH datasets is 42 (Additional File 7 Table S2). Despite the modest number of overlapping genes, the GO categories for the 720 genes with negative correlation between copy number and expression in the cancer data were strikingly similar to the RH genes with negative α and included categories of plasma membrane, signaling and receptor activity (Table 2). A Mann-Whitney rank test between TCGA and human RH GO scores was significant (*P *= 3.2 × 10^-6^).

**Table 2:** GO Enrichment for negative cis α in TCGA

Human	Score	FDR
**Biological Process**		

Multicellular Organismal Process	1.05 × 10 ^-12^	1.90 × 10 ^-9^

Cell-Cell Signaling	9.28 × 10 ^-11^	1.67 × 1^0 -7^

Cell Communication	4.33 × 10 ^-10^	7.81 × 1^0 -7^

Immune Response	6.04 × 10 ^-10^	1.09 × 10 ^-6^

Immune System Process	1.35 × 10 ^-9^	2.44 × 10 ^-6^

Response To Stimulus	5.88 × 10 ^-9^	1.06 × 10 ^-5^

Anatomical Structure Development	1.14 × 10 ^-8^	2.06 × 10 ^-5^

System Development	2.14 × 10 ^-8^	3.85 × 10 ^-5^

Organ Development	5.01 × 10 ^-8^	9.04 × 10 ^-5^

System Process	6.70 × 10 ^-8^	1.21 × 10 ^-4^

Transmission Of Nerve Impulse	1.32 × 10 ^-7^	2.39 × 10 ^-4^

Defense Response	1.72 × 10 ^-7^	3.10 × 10 ^-4^

Multicellular Organismal Development	2.42 × 10 ^-7^	4.37 × 10 ^-4^

Developmental Process	5.27 × 10 ^-7^	9.50 × 10 ^-4^

Signal Transduction	9.39 × 10 ^-7^	1.69 × 10 ^-3^

**Cellular Component**		

Intrinsic To Plasma Membrane	3.97 × 10 ^-22^	5.55 × 10 ^-19^

Integral To Plasma Membrane	5.78 × 10 ^-22^	8.08 × 10 ^-19^

Plasma Membrane	7.84 × 10 ^-20^	1.10 × 10 ^-16^

Plasma Membrane Part	1.21 × 10 ^-19^	1.69 × 10 ^-16^

**Molecular Function**		

Signal Transducer Activity	4.16 × 10 ^-12^	6.43 × 10 ^-9^

Molecular Transducer Activity	4.16 × 10 ^-12^	6.43 × 10 ^-9^

Receptor Activity	7.78 × 10 ^-10^	1.20 × 10 ^-6^

We sought confirmation of decreased *cis *effects on the X chromosome in TCGA data. We used male TCGA samples (N = 180) to exclude the effects of X chromosome inactivation (Figure 4C). However, similar conclusions were drawn from female TCGA data (N = 52, Figure 4D). In relation to the autosomes, mean gene expression on the X chromosome in the male TCGA samples showed a significant attenuation in response to increased copy number (Figure 4C). We divided X-linked and autosomal genes in the TCGA data into positively and negatively regulating *cis *ceQTLs and found that genes on the X chromosome possessed smaller effect sizes than the autosomes (paired t-test, 179 d.f., *P *< 2.2 X 10^-16 ^for both positive and negative). This is similar to what we observed in both human and mouse RH panels where effect sizes for genes on X were smaller in magnitude than autosomes (Figure 4D). Considering the selective pressure in cancer cells and the corresponding lack of uniform coverage compared to RH cells, overall, TCGA data is consistent with the findings of negative *cis *ceQTLs and the attenuated X-linked copy number/expression relationships in the RH datasets.

### *Trans *ceQTLs

There were a total of 17,347 *trans *loci at an FDR < 0.25. Of the 36,082 *trans *interactions between peak markers and genes in the human RH data, 39 have negative α (indicating repression) while the remaining 36,043 (99.9%) have positive α (induction).

Both the mouse and human RH datasets had genes regulated by multiple loci (Figure 3 horizontal marginal). To test for conservation of hotspots regulating multiple genes in *trans *(Figure 3 vertical marginal) in human and mouse, we remapped mouse ceQTLs onto the human genome using the UCSC Liftover utility. We then binned the human genome into 1 Mb bins and performed a chi-square test on the number of genes regulated by each bin in the two RH datasets. The result was not significant.

We then investigated the overlap of genes underlying *trans *ceQTLs between human and mouse RH data. For this analysis, we found the closest genes to regulating *trans *ceQTLs and counted the number of overlapping genes between the two species whenever orthologous genes could be identified. For regulating *trans *ceQTLs, 2,381 genes were found in mouse while 5,930 were found in human. The overlap of 1,745 was significant by chi-square test (P < 2.2 × 10^-16^).

We also examined the effect of *trans *ceQTLs regulating X chromosomal genes. The difference in effect sizes between autosomal and Xchromosomal loci was significant for positive α (P = 10^-2^) but much weaker than for *cis *ceQTLs. There were too few observations to test negative α (Figure 4D) on the X chromosome (N = 3). The X chromosome attenuation phenomenon appears to be specific for *cis *ceQTLs.

### *Trans *ceQTLs are functionally enriched

Contrary to a yeast eQTL dataset [16], *trans *ceQTLs in our original mouse RH dataset were enriched for GO categories related to transcription. We tested the enrichment of Gene Ontology categories using DAVID for 5,929 genes closest to a *trans *ceQTL in our human RH data at FDR < 0.25. The top 10 categories for the biological process ontology are displayed in Table 3 alongside enrichment results from our mouse RH dataset at the same threshold. A Mann-Whitney rank test on the GO category scores between these two datasets provides strong evidence of similarity (P = 4.9 × 10^-7^). Complete results are shown in Additional File 8 Table S3.

**Table 3:** Functional enrichment of *trans *ceQTLs at FDR < 0.25

Human	*P *value	FDR	Mouse	*P *value	FDR
**Biological Process**					

System Development	1.69 × 10^-25^	2.70 × 10^-22^	System Development	4.48 × 10^-15^	6.82 × 10^-12^

Anatomical Structure Morphogenesis	1.21 × 10^-18^	1.92 × 10^-15^	Organ Development	3.42 × 10^-13^	5.25 × 10^-10^

Cell Differentiation	2.96 × 10^-14^	4.73 × 10^-11^	Anatomical Structure Morphogenesis	6.17 × 10^-13^	9.47 × 10^-10^

Organ Development	7.90 × 10^-13^	1.26 × 10^-9^	Neuron Projection Development	2.36 × 10^-11^	3.63 × 10^-8^

Ion Transport	6.86 × 10^-12^	1.09 × 10^-8^	Axon Guidance	6.83 × 10^-11^	1.05 × 10^-7^

Cell-Cell Signaling	1.39 × 10^-11^	2.21 × 10^-8^	Cell Differentiation	1.17 × 10^-9^	1.79 × 10^-6^

Negative Regulation Of Biological Process	3.06 × 10^-11^	4.89 × 10^-8^	Negative Regulation Of Cellular Process	5.50 × 10^-9^	8.44 × 10^-6^

Regulation Of Multicellular Organismal Process	4.60 × 10^-10^	7.34 × 10^-7^	Cell Projection Morphogenesis	5.98 × 10^-9^	9.17 × 10^-6^

Cell Motion	5.61 × 10^-9^	8.96 × 10^-6^	Negative Regulation Of Biological Process	9.79 × 10^-9^	1.50 × 10^-5^

Negative Regulation Of Cellular Process	1.27 × 10^-8^	2.03 × 10^-5^	Cell Motion	1.28 × 10^-8^	1.97 × 10^-5^

Cell Morphogenesis	1.70 × 10^-8^	2.72 × 10^-5^	Cell Development	1.39 × 10^-8^	2.14 × 10^-5^

Transport	2.19 × 10^-8^	3.50 × 10^-5^	Cell Part Morphogenesis	2.78 × 10^-8^	4.26 × 10^-5^

Categories showing enrichment in the human RH data included signaling, development, binding, plasma membrane, and cytoskeleton. Remarkably, many of these same categories were enriched in the mouse dataset, showing conservation of function between the two species among *trans *ceQTLs, particularly ion related categories. Transcription factor related categories were enriched only in the mouse RH data at FDR < 0.25. However, transcription factor activity was enriched in the human RH data at FDR < 0.3.

### Regulatory loci in noncoding regions

At FDR < 0.25, a total of 1,128 out of 17,347 (6.5%) of *trans *ceQTLs mapped to noncoding regions of the human genome. We considered a ceQTL as noncoding if it was > 300 kb away from a known gene or microRNA according to UCSC's hg18 or mm7 gene location tables. The choice of a 300 kb cutoff is somewhat arbitrary, but it exceeds twice the - 2log_10 _*P *support radius (i.e., the width of the peak two -log_10 _*P *units from the maximum) used in this study.

**Figure 5:**
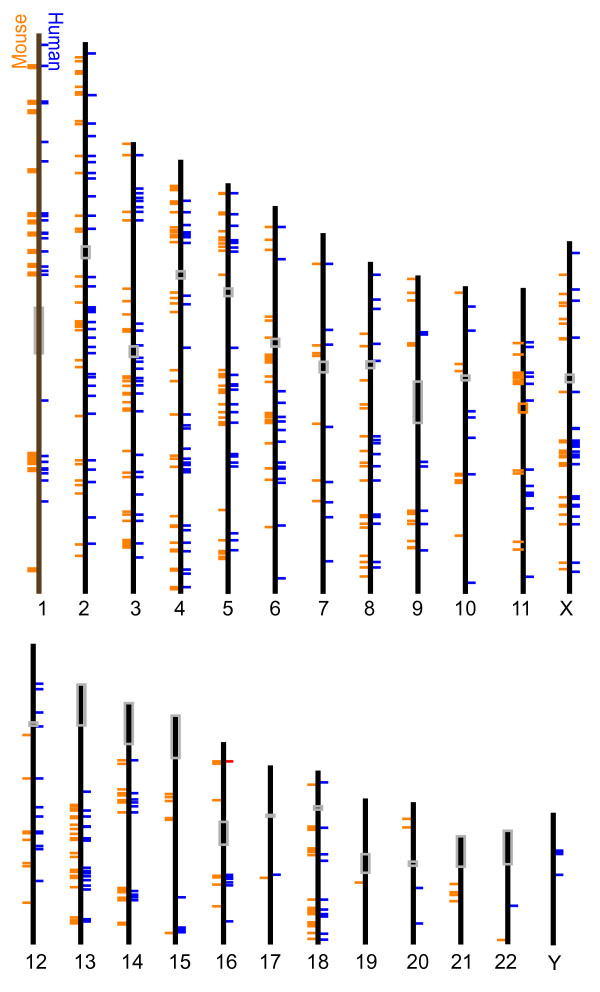
**Human and mouse noncoding ceQTLs**. Location of human (right) and mouse noncoding ceQTLs mapped onto the human genome (left). Centromeres are grey boxes.

Assuming that closely linked ceQTL peaks represent individual loci regulating multiple genes, we merged noncoding ceQTLs if the peaks were < 300 kb from each other (Methods). At FDR < 0.25, there were 325 noncoding ceQTLs in human and 370 in mouse. Since some noncoding ceQTLs in mouse map to more than one noncoding ceQTL in human (and vice versa), we enforced a rigorous one-to-one mapping of mouse noncoding blocks to human noncoding blocks resulting in 369 possible noncoding blocks in common between the two species. Chi-square analysis of the number of shared blocks containing ceQTLs (118) between mouse and human (205 and 199, respectively) between the two species was not significant. At a more liberal threshold of FDR < 0.3, the overlap of noncoding blocks was significant (P = 10^-7^). Figure 5 shows the co-localization of mouse and human noncoding ceQTL blocks on the human genome.

We applied Gene Set Enrichment Analysis [17] to the genes regulated by the eight syntenic noncoding ceQTLs with the highest -log_10 _*P *values in both mouse and human datasets (-log_10 _*P *> 4). No pair of mouse-human gene lists regulated by a common ceQTL had an overlap in their enriched GO categories. However, we found one noncoding ceQTL located on the mouse X chromosome at 20.6 Mb and the syntenic region of the human × chromosome at 115.9 Mb that affected expression of an overlapping set of gene targets regulated by 19 microRNAs (χ^2 ^= 8.74, 1 d.f., *P *= 3.1 X 10^-3^). The noncoding ceQTL itself did not harbor any microRNAs according to MiRscan (see below).

### microRNAs in noncoding regions

The existence of noncoding *trans *ceQTLs suggested there may be unknown genomic elements in those regions. One possibility included unidentified microRNAs. We used MiRscan [18] to screen the positionally conserved noncoding ceQTLs with FDR < 0.25. No regions resulted in significant MiRscan scores.

### Known noncoding elements do not explain noncoding ceQTLs

Several recent reports using next-generation RNA-Seq and ChIP-Seq methods have found evidence of novel genes and functional RNAs in noncoding regions, illuminating the role of "dark DNA". We examined the positional overlap of three such datasets. The first was a deep RNA-Seq study of the mouse transcriptome which revealed evidence of novel genes [19]. In a ChIP-Seq study, a new class of large intervening noncoding RNAs (lincRNA) was identified due to the preferential association of histone H3 trimethylated at either lysine4 or lysine36 with these elements [20]. Ultraconserved regions [21] are noncoding regions > 200 bp perfectly conserved across multiple species. They possess no known function, yet appear to be under purifying selection.

**Figure 6:**
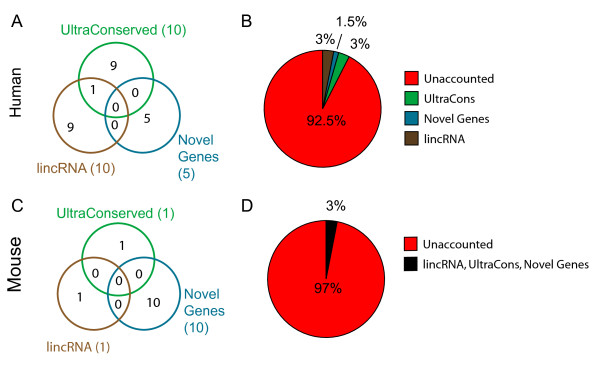
***Trans *ceQTLs in noncoding regions are not explained by recently discovered functional elements**. (A) Venn diagram of the number of noncoding ceQTLs in human at FDR < 0.25 that can be accounted for by lincRNAs, novel genes and ultra conserved regions. For example, 10 noncoding ceQTL blocks contain lincRNAs, one of which overlaps with noncoding ceQTL blocks containing ultra conserved regions. (B) The majority of the 325 noncoding ceQTL blocks in human at FDR < 0.25 do not contain lincRNAs, novel genes or ultra conserved regions. (C) Number of noncoding ceQTL blocks in mouse at FDR < 0.25 that can be explained by recently discovered genomic elements. (D) Of 370 noncoding ceQTLs in mouse, 3% can be accounted for by recently discovered functional elements.

The overlap between these three classes of newly discovered functional elements and our human noncoding ceQTLs at FDR < 0.25 was sparse (4% overlap; 96% of noncoding ceQTL blocks unexplained) (Figure 6A, B). Only 3.2% of the mouse RH noncoding ceQTL blocks overlap with these same elements (Figure 6C, D). Although we used a relatively liberal FDR < 0.25 to identify the > 320 noncoding ceQTLs, the number of true noncoding ceQTLs is still expected to be ~240. This greatly exceeds the number of lincRNAs, ultraconserved regions and novel genes, suggesting these elements cannot be the regulatory elements underlying most of our noncoding ceQTLs. The -log_10 _*P *values of the *trans *noncoding ceQTLs closest to either lincRNAs or novel genes were among the lower scoring (Additional File 9 Figure S6), implying that the majority of the noncoding ceQTLs represent novel but still undefined biological regulators.

While enhancers are known to affect gene expression at a distance, none of the non-coding ceQTLs can represent these regulatory elements. Unlike meiotic mapping, a breakpoint in RH mapping physically separates a regulatory element from its corresponding gene. The element is instead placed next to a randomly selected gene in each RH clone and will not act as a consistent *trans *regulatory locus.

### Comparison of RH data with normal tissues

In order to evaluate our artificial human RH system against an *in vivo *biological data set, we compared the gene expression from the RH experiments to the human Novartis SymAtlas [22], a compendium of gene expression across multiple tissues. Using a common set of 12,368 genes, we constructed a correlation matrix of expression for gene pairs across the RH panel and a similar matrix across the 79 tissues of the SymAtlas. We then subtracted the two matrices and computed the Frobenius norm (Methods) to quantify the distance between the two data sets. To generate a null distribution, the gene expression values from the RH data were permuted, a new correlation matrix was computed and subtracted from the SymAtlas correlation matrix and the Frobenius norm recomputed. Of 10,000 permutations, none showed a score smaller than the observed score (*P *< 10^-4^) (Additional file 10 Figure S7). This result suggests that pair-wise gene expression changes obtained from copy number variation in the RH panel are similar to those obtained from regulated gene expression in multiple tissues of a mammalian organism.

## Discussion

The relationship between copy number and gene expression has only begun to be explored as most studies are focused on identifying regions of copy number variation (CNV) [23-25]. The first studies to extensively explore CNV effects on expression in mice highlighted the potential for widespread impact of CNVs on shaping the transcriptome of various tissues [6,26]. Recent studies of CNV effects on gene expression in human and mouse rely upon naturally occurring variation (deletions, duplications, triplications, etc) and have been limited to *cis *effects [27,28]. Radiation hybrid panels allow a genome-wide survey of gene expression changes due to copy number increases and are not limited to regions of previously identified CNVs.

Several lines of evidence support the broader applicability of RH panels in understanding gene expression networks. Though highly multiplexed, RH panels are not unlike other systems such as transgenic organisms or transfected cell lines which have given useful biologically insights. Phenotypic mapping experiments using radiation hybrids have successfully located human and murine viral entry proteins [29-32] by exploiting the ability of RH clones to correctly express exogenous genes and synthesize and post-translationally modify the resulting proteins. Recent sequencing efforts of the hamster genome showed that coding sequences are 88% conserved with human [33].

The gene-gene correlation between human RH and SymAtlas datasets also implies no substantial difference in gene expression between our human RH panels and *in vivo *gene expression for the 12,000 genes we tested. One caveat is their different sources of genetic variation so this result should be considered in context with other available evidence. Unlike genetic coexpression studies, the high resolution of the RH approach allowed construction of directed genetic networks from the mouse RH data. These directed networks showed significant overlap with other networks including protein-protein interaction and coexpression networks [34]. Adding the human RH data will improve the resolution and power of the directed RH genetic networks giving additional insights into the hierarchical circuitry of gene regulation.

Using a human-hamster RH panel, a mouse-hamster RH panel, an aneuploid mouse dataset and publicly available TCGA data, we present strong evidence that many genes possess the ability to decrease their gene expression in response to increased copy number (i.e., possess negative *cis *α). In the mouse and human RH datasets, 30% of genes show this ability compared to 6% of surveyed TCGA genes. Some of this is likely due to the difference in coverage: the entire human/mouse genome was represented in the RH panels while only 38% of the genome was covered in TCGA data. A small number of negative *cis *alphas have been reported in human [5,8,27] and mouse [26,28], but the RH approach is the first to interrogate the entire mammalian genome.

Additional factors may underlie some of these negative cis ceQTLs, but are unable to account for the totality of negative *cis *ceQTLs. Antisense transcription plays no significant role and the inclusion of partial length genes in each RH clone could maximally account for only a minority (< 21%) of negative *cis *ceQTLs.

Across the RH and TCGA data, the most enriched gene ontology categories for genes that decrease expression in response to increased copy number involved signaling, receptor activity and membrane functions. This finding is new and suggests that signaling pathways are tightly regulated and may possess autoregulatory feedback to compensate for increased copy number. Signaling genes were recently found to be enriched among human CNVs [24] and under positive selective pressure [35], possibly because negative cis α values confer a regulatory robustness in the face of sequence changes. Study of individual genes should reveal details of the responsible mechanisms.

We found 42 common genes with negative cis α between the two RH and TCGA data sets (Additional file 2 Table S2). Surprisingly, the relatively modest overlap in the number of genes still yields a high degree of similarity in GO categories across the three data sets suggesting conserved pathways are affected.

We observed that *cis *ceQTLs on the human X chromosome showed substantially lower effect sizes than autosomes - a discovery we first noted in the mouse RH panel. The attenuation of the relationship between dosage and expression is independent of *Xist *mediated X chromosome inactivation and may represent a form of previously unseen dosage compensation in mammals. In placental mammals, X chromosome inactivation occurs through the expression of *Xist*, a noncoding RNA on the future inactive X chromosome (Xi) [36]. Transcribed sequences from the *Xist *locus coat the Xi-elect by binding nongenic regions of the X chromosome [37,38]. The predicted secondary RNA structure of *Xist *possesses two stem loops and may serve as a scaffold for silencing factors [39]. Chromatin modification [40], scaffold proteins [41], and polycomb proteins [42] have all been implicated in the initiation and maintainance of X chromosome inactivation although the picture is far from clear. In contrast to mammals, *Drosophila *[43] and *C. elegans *[44] both use transcriptional control for X chromosome dosage compensation. The autoregulatory control of X chromosome expression found in the human and mouse RH panels may thus represent an evolutionary remnant of these invertebrate dosage compensation mechanisms which has since been supplemented by X chromosome inactivation. The same attenuation pattern was found in male TCGA data on the X chromsosome. While cancer resembles RH clones in some respects, cancer cells differ in several important aspects such as mutation, selection, heterogeneity of fragment length and differences in genome coverage.

Among *trans *loci, we found evidence of conserved regulating genes between the human and mouse RH panels. *Trans *ceQTLs were particularly associated with genes involved in binding, signaling and ion-channel activity suggesting that these genes tend to represent network hubs and that copy number changes in these genes can contribute to non-lethal variation. We found enrichment of transcription factor activity in mouse but not human RH data at FDR < 0.25. However, at FDR < 0.3, transcription factor activity was enriched in human RH as well. *Trans *regulatory hotspots have been observed in eQTL studies involving yeast [16], mouse [45] and human [46] and are commonly interpreted as evidence for master regulators. However, unanticipated factors in the data may contribute to false positives. For instance, a high degree of relatedness between mouse strains has produced signatures of regulatory hotspots [47] and association with groups of highly correlated genes has produced unlikely regulatory hotspots [48,49]. Integrating additional information such as transcription factor binding sites, protein-protein interaction data and functional analysis is helpful in identifying likely candidates when unanticipated heterogeneity may exist [48,50].

We found noncoding ceQTLs in both human and mouse. Debate continues about the importance of the substantial portion of the genome that does not code for genes. While it is clear that much of the genome is actively transcribed, the role of these regions is unclear. We examined new datasets containing genes and functional genomic elements in noncoding regions, yet the vast majority of our noncoding ceQTLs cannot be explained by these recent discoveries. We also found no significant overlap of the location of noncoding ceQTL blocks in both species at FDR < 0.25. At a slightly less stringent FDR < 0.3, there is significant overlap in the locations of noncoding ceQTL blocks in both species but the regulated genes differ. This may reflect evolutionary divergence. Indeed, microRNAs, many of which are conserved across species, have also been found to show species-specific regulation [51].

Our own search for novel microRNAs in noncoding ceQTLs yielded no candidates, though it is likely that improved screening techniques and computational algorithms may aid their discovery. Also, there were very small numbers of other unconventional RNAs such as linc RNAs in the noncoding ceQTLs. Thus, unanticipated forms of gene regulation seem likely. While the RH approach does not reveal possible mechanisms of action, the noncoding ceQTL data could act as a guide for discovery of these novel elements by allowing transfection of overlapping genomic DNA fragments traversing the ceQTL combined with transcript profiling as a bioassay.

Radiation hybrid panels exist for a number of other organisms including sheep [52], pig [53], cow [54,55], rat [56] and dog [57]. The potential exists for probing species-specific copy number effects on gene expression. Amalgamating these data sets can also be used to improve mapping resolution and examine common networks of gene regulation and regulatory regions.

## Conclusions

Radiation hybrid panels are a valuable tool for probing the relationship between copy number and gene expression in the mammalian genome in a largely unbiased manner. In both human and mouse radiation hybrid panels, we have mapped to high resolution *cis *and *trans *loci capable of affecting gene expression due to copy number change and found a number of consistent results. Approximately 30% of genes show an inverse correlation between increased copy number and gene expression and genes on the X chromosome show an attenuated response to copy number increase as compared to autosomes, suggesting a potentially novel form of dosage compensation. Copy number perturbations of noncoding regions were shown to affect gene expression as well and the lack of known control elements in these regions may imply novel regulatory loci.

## Methods

### Cells

RH clones were thawed and cultivated in alpha-MEM with 10% FBS, 1X ampicilin and 1X HAT. Cells were trypsinized and DNA and RNA harvested as described in our previous study [8].

### Microarray analysis

RNA from each of the 80 available radiation hybrid clones and A23 recipient hamster cell line was hybridized in duplicate (technical replicates) to single channel Illumina HumanRef-8 v1.0 BeadChips by the UCLA Southern California Genotyping Consortium according to manufacturer's protocols. The raw data was extracted using Illumina BeadStudio v1.5.1 and median normalized using Genespring GX (Agilent). Duplicate array measurements were log averaged prior to the construction of RH to A23 ratios for 20,996 genes.

The average correlation between replicates was r = 0.92 (*P *< 2.2 × 10^-16^). Hierarchical clustering always grouped duplicate arrays together (Additional File 2 Figure S2).

### Comparative Genomic Hybridization

DNA from each radiation hybrid clone was extracted and hybridized to Agilent 244 K human comparative genomic hybridization (aCGH) arrays which contain 60 mer oligonucleotide probes. Hamster A23 DNA, serving as the control, was the other channel. Arrays were labeled and scanned according to manufacturer's instructions.

### Normalization of aCGH data

Preprocessing and normalization of the raw aCGH data was performed as described previously [8]. Briefly, raw aCGH intensity data (RH/A23) for 235,829 markers was log_10 _transformed and then averaged over a sliding window of ten adjacent markers for each cell line. The bimodal distribution of log_10 _intensity across all cell lines (Additional File 11 Figure S8A-B) showed markers with no copy number increase and those with an increase of one or more copies. Most copy number changes were an increase of one with the probability of retaining two copies ~1%.

Since the recipient hamster cells are male, the copy number increase for the autosomes was three compared to two and two compared to one for the sex chromosomes. We therefore normalized the log_10 _transformed aCGH data by centering the first mode at zero (log_10 _(2/2)) and then scaling the data so that the second mode was centered at log_10_(3/2) for the autosomes and log_10_(2/1) for the sex chromosomes.

To quantify marker loss/gain compared to the legacy PCR data as a result of passaging of G3 radiation hybrid clones, we averaged the log_10_(RH/A23) aCGH ratio for the ten markers closest to a STS marker. If this value exceeded the 95^th ^percentile of the first mode of the omnibus distribution, we classified this region as retained. Of the 80 available G3 clones, one clone did not match any PCR genotypes and was excluded from all subsequent analyses.

### Linear model

We used a linear model to characterize the change in gene expression due to increased copy number [8]. For each gene, we modeled the data as *y *= μ + α*x *where *y *is the normalized log_10_(RH/A23) expression, *x *is the log_10 _(RH/A23) aCGH data, μ is the baseline gene expression and *α *is the ordinary least squares estimate reflecting the effect size. This model was compared with a reduced model *y *= μ exactly like an F-test, except that permutation was used to generate a null distribution of residuals and assign P values. We tested 20,996 genes and 235,829 markers, to calculate all 4,951,465,684 possible combinations.

The permutation employed random re-assortment of the gene expression data and recalculation of the F-statistic five times for each combination (5 × 20,996 × 235,829). The correlation structure of the markers was retained between each permutation. The pooled F-statistics served as the empirical null and *P *values were calculated as the frequency of null values greater than the observed F statistic.

The Benjamini-Hochberg method was used to control false discovery rates (FDRs). Since *cis *ceQTLs (marker < 5 Mb from a gene) test a different set of hypotheses than *trans *ceQTLs (marker >5 Mb from a gene), we applied FDR separately to *cis *and *trans *ceQTLs.

We also utilized the R/Bioconductor package DNAcopy to bin CGH data into 0 or 1 extra copies of the donor locus in order to assess species specific hybridization artifacts. Copy number, instead of CGH intensity, was then used in the linear model. A comparison of *α*'s obtained by our original procedure and using the binned CGH data showed excellent concordance (r = 0.95, P < 2.2 × 10^-16^) (Additional File 12 Figure S9).

### TCGA data analysis

We downloaded matched aCGH and expression data (N = 237) from The Cancer Genome Atlas (TCGA, http://tcga.cancer.gov) glioblastoma multiforme data portal. These data were normalized by the TCGA consortium. TCGA aCGH data consists of a tumor sample hybridized to one channel and a male reference sample hybridized to the other channel. Because only 38.8% of autosomes and 68% of the X chromosome are affected by copy number perturbation in this data set, we discarded aCGH markers that did not show a change in copy number (e.g., > log_2 _(3/2) or < log_2 _(1/2) for autosomes) in at least one sample. For each CGH marker within a 5 Mb radius, we performed linear regression to estimate the effect of increased copy number on gene expression. An FDR correction was applied to the data such that all TCGA results have a FDR < 0.05 (*P *< 0.04).

### Comparison of *cis *ceQTL overlap between data sets

To compare the overlap of ceQTLs with positive and negative cis α between human and mouse RH data, we performed a chi-square test of a 2 × 2 contingency table in R. Orthologous genes between the two data sets were first identified and then we counted the number of *cis *ceQTLs with positive and negative α in both data sets as well as those that those that were positive in one data set and negative in the other. Comparison of the positive-positive and negative-negative cells of the 2 × 2 table with the expected counts indicated enrichment. The same procedure was used for comparing human RH with mouse trisomic and TCGA data.

### Evaluation of hamster transcripts on human microarrays

Exploiting the high conservation of coding sequences in mammals, we used a human microarray platform to interrogate the expression of the donor human and recipient hamster genes in the G3 RH panel. Illumina BeadChip probes consist of relatively long (50 mer) oligonucleotides potentially allowing evaluation of transcripts from both species.

We tested whether the expression arrays could detect hamster and human transcripts with comparable efficiency. RNA extracted from hamster and human liver, kidney and heart and compared the relative expression signals as a ratio (Additional File 1 Figure S1A). The bulk of the ratios were centered around zero for log_10 _(human/hamster) expression, indicating equivalent performance in measuring hamster and human expression for most genes. As expected, some probes showed a preference for human transcripts.

While species differences in sequence hybridization may influence detection of *cis *ceQTLs, detection of *trans *ceQTLs is not directly affected by such variation. We therefore compared the distributions of log_10 _human/hamster tissue gene expression for *cis*- and *trans*-regulated genes, with the *trans *ceQTL distribution acting as a control. The difference in expression ratios between the two groups was not large (Additional File 1 Figure S1B-D). As expected, there was some preference for human genes (18.8%). Based on this evidence, most of our *cis *ceQTLs (>80%) are not due to differences in hybridization on the microarray.

To evaluate array CGH use for hamster, we co-hybridized genomic DNA from hamster and human to the array and evaluated the signal intensities for each channel separately. Correlation between the two species on the aCGH array was 0.57 and the means of the human and hamster signals are quite close (6.7 and 7.0 respectively). For human, the signal intensity has a larger standard deviation than hamster (0.88 versus 0.37 respectively) (Additional File 1 Figure S1E-F). Preferential binding of human DNA would lead to a conservative bias as it minimizes signal from the hamster genome which is expected to be unperturbed.

### *Trans *Hotspot FDR

We calculated the probability that a marker would regulate more than *n *genes using a Poisson distribution with mean equal to the average number genes regulated by *trans *ceQTLs across all markers. These p-values were then subjected to Benjamini-Hochberg correction to obtain an FDR. Similarly, the probability that a gene is regulated by more than *m *ceQTLs was modeled as Poisson with mean equal to the average number of markers regulating each gene. These p-values were FDR corrected as above.

### Noncoding regions

We defined a *trans *ceQTL as noncoding if > 300 kb away from a known gene or microRNA using the UCSC human hg18 (NCBI 36.1) or mouse mm7 (NCBI 35.1) gene and microRNA tables. The UCSC gene set is larger (~50,000 entries) and less conservative than RefSeq (~20,000 entries), including genes with alternative start sites, alternatively spliced exons and putative but unknown genes. CeQTL peaks in noncoding regions are likely due to the same genes if nearby and were merged together if within 300 kb in both human and mouse datasets.

### Conversion of mouse locations to human locations

UCSC's LiftOver utility allows the conversion of genome coordinates from one species to another, using whole genome alignments (nets and chains) generated by their BLASTZ analysis [58,59]. The 232,626 mouse markers were subjected to the recommended minMatch parameter of 0.10 for interspecies conversion using mm7 to hg18 liftover chain files. Approximately 160,000 markers were converted at this level.

### microRNA discovery

We essentially followed the published methodology for using MiRscan [18]. First, we employed the RNAfold program from the Vienna RNA software package [60,61] and scanned 100 nt windows in our mouse noncoding regions to find regions of stable hairpin formation. As a cutoff, we used a minimum free energy value of -25 kcal/mol. All candidate regions were BLASTed against human noncoding regions to find the best matching region, which was then fed to RNAfold to determine their minimum hairpin free energy. Regions meeting the same minimum free energy value of -25 kcal/mol were passed to MiRscan, which uses multiple conservation criteria to score a region for possible microRNA content. Of the ~325 noncoding regions tested, none were significant.

### Comparison with SymAtlas

The Novartis SymAtlas [22] contains expression values for ~22,000 probes across 79 different human tissues. A common set of 12,368 genes were identified between the SymAtlas and the human RH data. Two separate gene-gene correlation matrices were constructed based on the expression data, one each for RH and SymAtlas. We then subtracted the two correlation matrices from each other to obtain the matrix *A *and computed the Frobenius norm defined as

$${∥A∥}_{F}=\sqrt{\underset{i}{\sum }\underset{j}{\sum }{\left|a_{ij}\right|}^{2}}$$

which serves as a distance measurement between the two correlation matrices. A low score represents high similarity. To generate a null data set, we permuted the assignment of expression values in the human RH data, created a new correlation matrix, subtracted the matrix from the SymAtlas matrix and recomputed the Frobenius norm 10,000 times. The *P *value is determined by the number of times a permuted score is less than our observed score. The gene expression correlation structures were preserved in the permuted matrices.

### Data Availability

Expression microarray and array comparative genomic hybridization (aCGH) data were submitted to the Gene Expression Omnibus under accession number GSE19003.

### Software

To automate, parallelize and optimize the computational analysis, custom Perl and C programs and modules were written to handle data and manage applications. Standalone BLAST was used to create custom sequence databases and Bioperl packages [62] used to automate BLAST queries and manipulate sequence data. BLAT [63] was used to obtain genome coordinates for microarray probes.

## Authors' contributions

R.T.W., A.H.K., C.C.P. carried out experiments. R.T.W., S.A., K.L. analyzed data. R.T.W., D.J.S. wrote the paper. D.J.S. designed research. All authors have read and approved the final manuscript.

## Supplementary Material

Additional file 1 **Figure S1**. Evaluation of human microarrays. (A) Log_10 _(human/hamster) expression ratios averaged across kidney, heart and liver. (B) Log_10 _(human/hamster) expression ratios for genes regulated by both *cis *(pink) and *trans *(blue) ceQTLs. The overlap between the two distributions is purple. (C) Log_10 _(human/hamster) expression ratios for genes regulated by *cis *ceQTLs. (D) Log_10 _(human/hamster) expression ratios for genes regulated by *trans *ceQTLs. (E) aCGH signal distribution for hamster and (F) human. Click here for file

Additional file 2 **Figure S2**. Expression arrays showed good replicability. Hierarchical clustering of expression arrays always placed duplicates next to each other. Duplicates referred to as 'a' and 'b'. Click here for file

Additional file 3 **Figure S3**. Retention frequency based on aCGH data. (A) aCGH intensity data for human RH clone 12 along chromosome 2 matches historical PCR data well (red lines) but does show some loss. (B) Retention frequency of human donor genome across all 79 RH clones. Solid line is loess smoothed with parameter 0.02. (C) Retention frequency of chromosome 6 is relatively uniform except for the centromere (grey) which shows preferential retention. (D) The *Tk1 *gene (red arrow) is retained at 100% as expected for the selectable marker. (E) The difference in retention frequency between centromeric (grey bars) and noncentromeric (red bars) region for all chromosomes is statistically significant (Welch's *t *> 8.1, d.f. > 477, *P *< 10^-15^). (F) The X chromosome has ~50% retention frequency of the autosomes because the donor cell line was male. The Y chromosome has an apparently higher retention frequency than the X, probably because the Y has a proportionally higher percentage of centromeric sequence (cf. Figure S4E). Error bars s.e.m. Click here for file

Additional file 4 **Figure S4**. Mapping resolution and effect sizes. (A) The median distance between a human gene and its *cis *ceQTL at FDR < 0.4 is 531 kb. (B) Distribution of human *cis *ceQTL α values. Positive α indicates induction of gene expression due to copy number increase, while negative α indicates repression. Click here for file

Additional file 5 **Table S1**. GO Enrichment for positive cis α at FDR < 0.25. Click here for file

Additional file 6 **Figure S5**. Comparison of genes with positive and negative cis α. (A) Histogram of expression values for genes with positive cis α (pink) and negative cis α (blue) with means 12.04 and 11.99 respectively. The overlap is in purple. (B) Occurrence of full length genes across all 79 RH clones. Each gene is found in its entirety on average 6 times. 3,422 genes are never found in their entirely across all clones. (C) Scatterplot of cis α's derived from the peak marker and its neighbor (r = 0.99, P < 2.2 × 10^-16^). (D) Cis α's of the peak marker and the 5^th ^closest marker (~75 kb away). Correlation is 0.96 (P < 2.2 × 10^-16^). Click here for file

Additional file 7 **Table S2**. Common genes with negative *cis *α in mouse RH, human RH and TCGA. Click here for file

Additional file 8 **Table S3**. Functional enrichment of *trans *ceQTLs at FDR < 0.25. Click here for file

Additional file 9 **Figure S6**. Distribution of -log_10 _*P *values for ceQTLs in human noncoding regions. Noncoding ceQTLs closest to known lincRNAs and recently discovered unconventional genes are indicated by red arrows and tend to be among the lower -log_10 _*P *values. Click here for file

Additional file 10 **Figure S7**. Comparison between human RH and SymAtlas. Distribution of Frobenius norm values for distance between human RH and SymAtlas data using permuted expression values. Observed human RH-SymAtlas distance shown in red. Units are arbitrary. Click here for file

Additional file 11 **Figure S8**. Log_10 _(RH/A23) aCGH intensity data is bimodal. (A) Histogram of aCGH intensity for all RH clones. The large mode represents equivalent copy number between RH clones and hamster A23 control genomes, while the smaller mode to the right indicates markers with an extra copy in the RH clones. (B) Close up view of the second mode. Click here for file

Additional file 12 **Figure S9**. Comparison of α from binned and continuous CGH data. CGH data was either binned into 0 or 1 extra copies or used as continuous values and used to calculate α. The correlation is 0.95, P < 2.2 × 10^-16^. Click here for file
