# Supplementary figures and images for: Effects of genome-wide copy number variation on expression in mammalian cells
Source: BMC Genomics. 2011 Nov 16;12:562. doi: 10.1186/1471-2164-12-562 (PMC3287593; doi:10.1186/1471-2164-12-562)

FIGURE S1

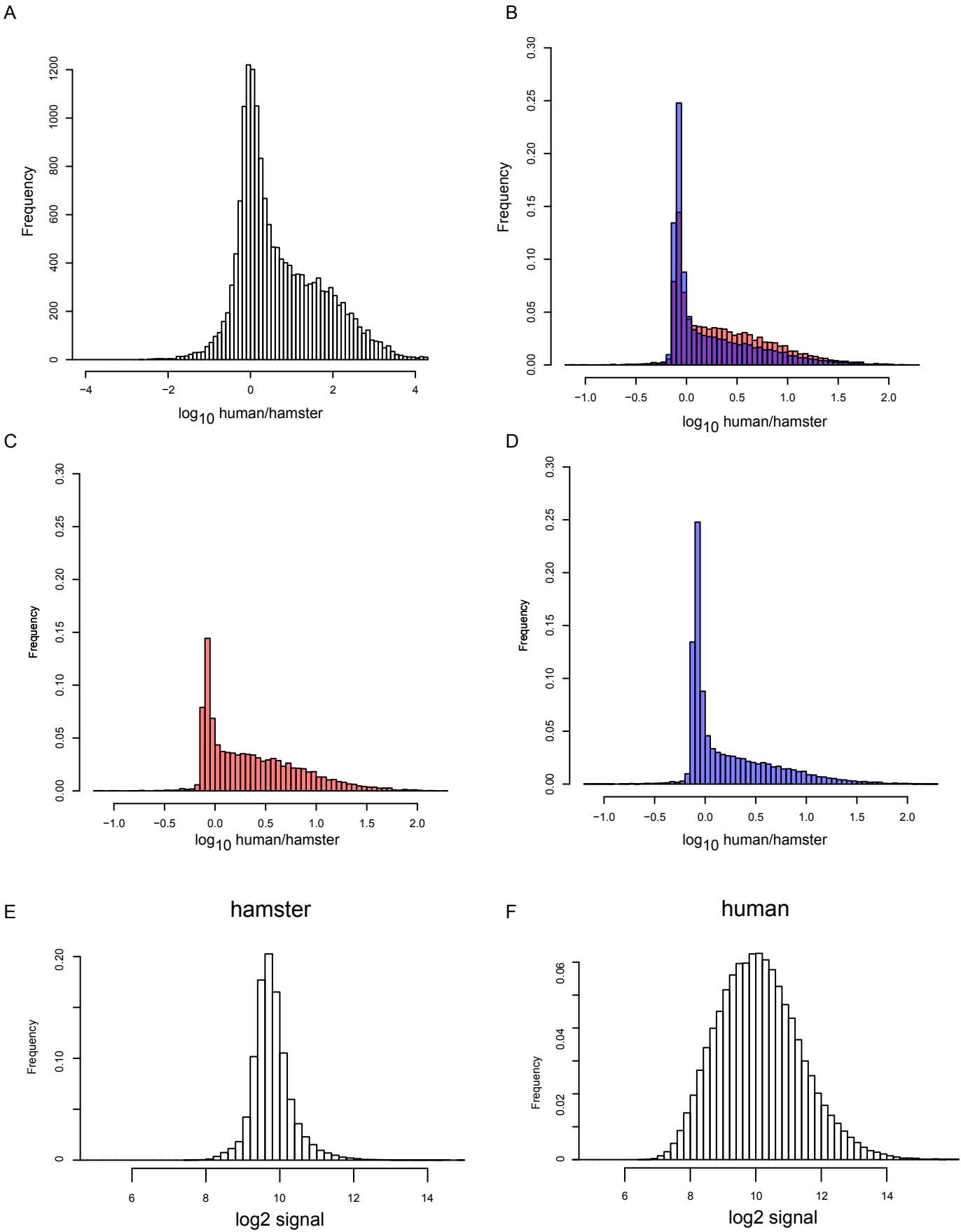

Supplement: Additional file 1 — Figure S1. Evaluation of human microarrays. (A) Log10 (human/hamster) expression ratios averaged across kidney, heart and liver. (B) Log10 (human/hamster) expression ratios for genes regulated by both cis (pink) and trans (blue) ceQTLs. The overlap between the two distributions is purple. (C) Log10 (human/hamster) expression ratios for genes regulated by cis ceQTLs. (D) Log10 (human/hamster) expression ratios for genes regulated by trans ceQTLs. (E) aCGH signal distribution for hamster and (F) human. [file 1471-2164-12-562-S1.PDF]

FIGURE S2

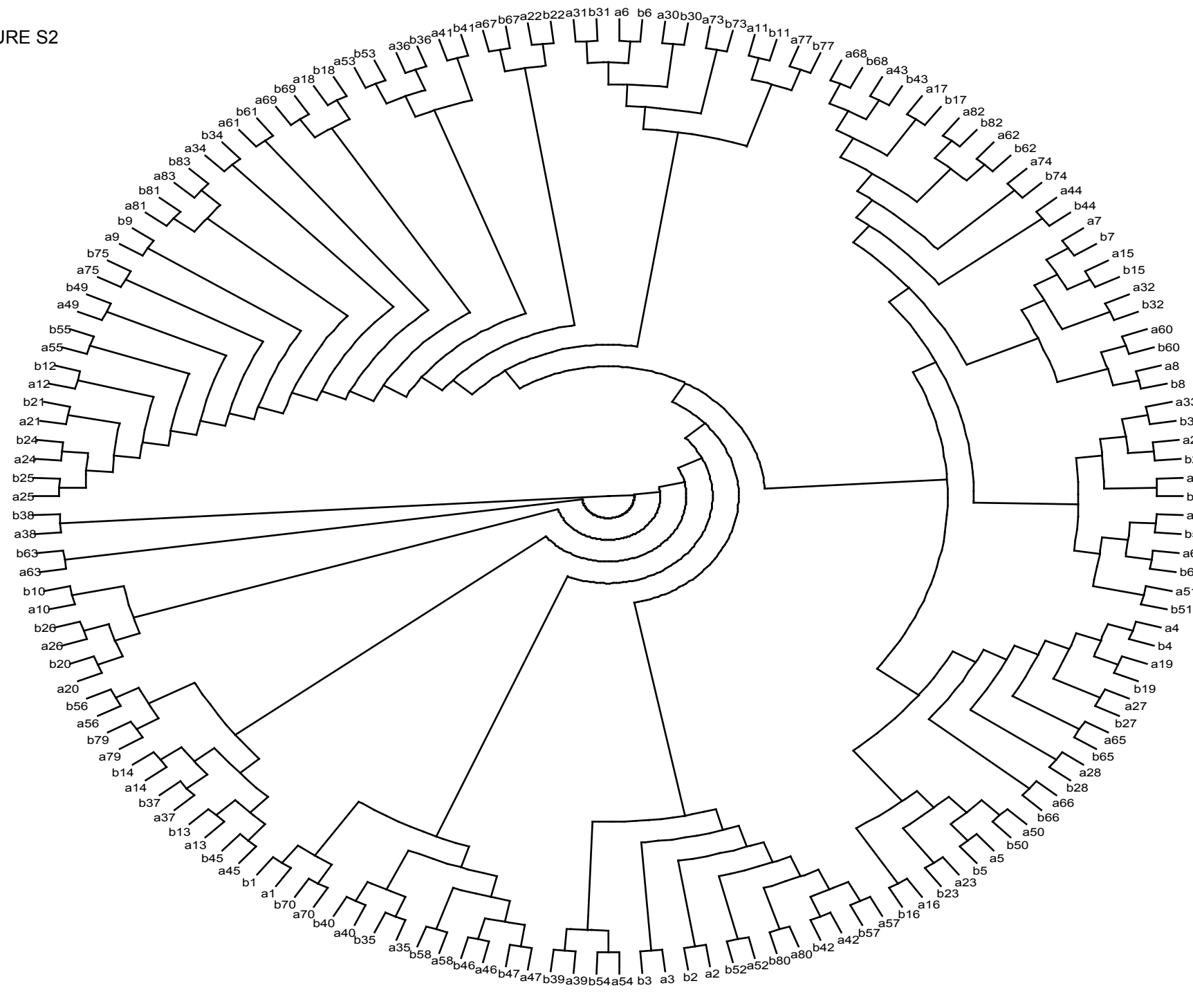

Supplement: Additional file 2 — Figure S2. Expression arrays showed good replicability. Hierarchical clustering of expression arrays always placed duplicates next to each other. Duplicates referred to as 'a' and 'b'. [file 1471-2164-12-562-S2.PDF]

FIGURE S3

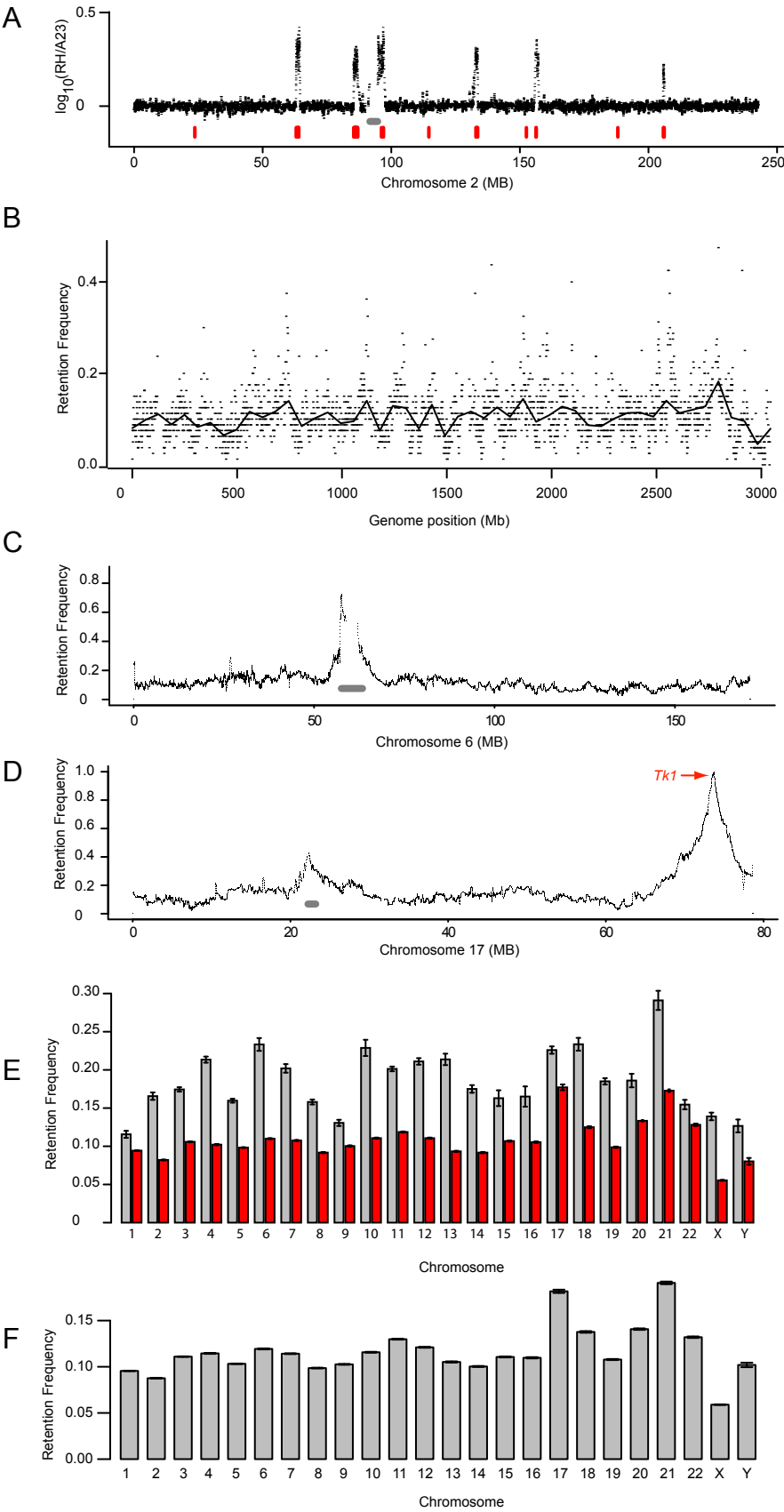

Supplement: Additional file 3 — Figure S3. Retention frequency based on aCGH data. (A) aCGH intensity data for human RH clone 12 along chromosome 2 matches historical PCR data well (red lines) but does show some loss. (B) Retention frequency of human donor genome across all 79 RH clones. Solid line is loess smoothed with parameter 0.02. (C) Retention frequency of chromosome 6 is relatively uniform except for the centromere (grey) which shows preferential retention. (D) The Tk1 gene (red arrow) is retained at 100% as expected for the selectable marker. (E) The difference in retention frequency between centromeric (grey bars) and noncentromeric (red bars) region for all chromosomes is statistically significant (Welch's t > 8.1, d.f. > 477, P < 10-15). (F) The X chromosome has ~50% retention frequency of the autosomes because the donor cell line was male. The Y chromosome has an apparently higher retention frequency than the X, probably because the Y has a proportionally higher percentage of centromeric sequence (cf. Figure S4E). Error bars s.e.m. [file 1471-2164-12-562-S3.PDF]

FIGURE S4

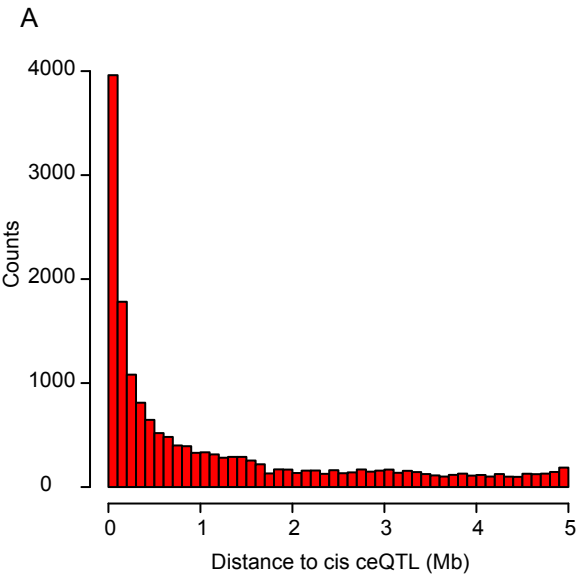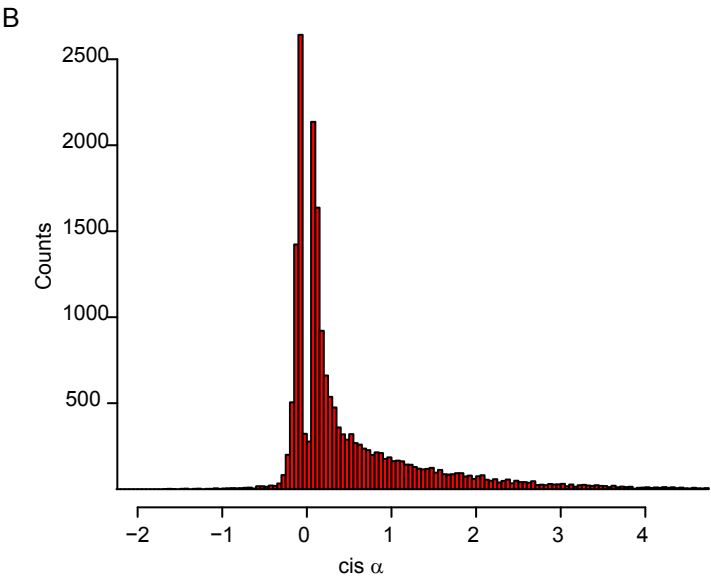

Supplement: Additional file 4 — Figure S4. Mapping resolution and effect sizes. (A) The median distance between a human gene and its cis ceQTL at FDR < 0.4 is 531 kb. (B) Distribution of human cis ceQTL α values. Positive α indicates induction of gene expression due to copy number increase, while negative α indicates repression. [file 1471-2164-12-562-S4.PDF]

FIGURE S5

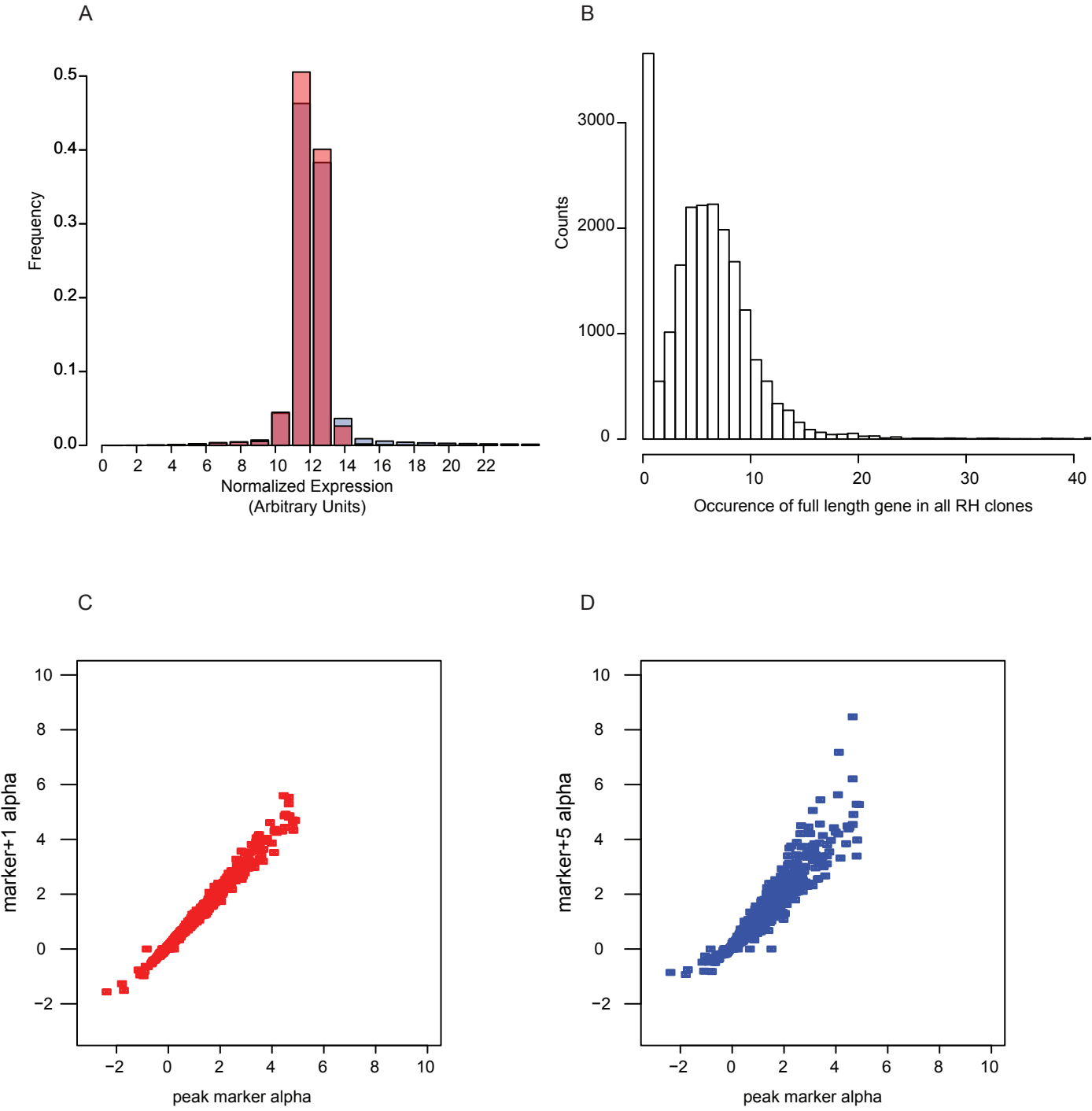

Supplement: Additional file 6 — Figure S5. Comparison of genes with positive and negative cis α. (A) Histogram of expression values for genes with positive cis α (pink) and negative cis α (blue) with means 12.04 and 11.99 respectively. The overlap is in purple. (B) Occurrence of full length genes across all 79 RH clones. Each gene is found in its entirety on average 6 times. 3,422 genes are never found in their entirely across all clones. (C) Scatterplot of cis α's derived from the peak marker and its neighbor (r = 0.99, P < 2.2 × 10^-16^). (D) Cis α's of the peak marker and the 5th closest marker (~75 kb away). Correlation is 0.96 (P < 2.2 × 10^-16^). [file 1471-2164-12-562-S6.PDF]

FIGURE S6

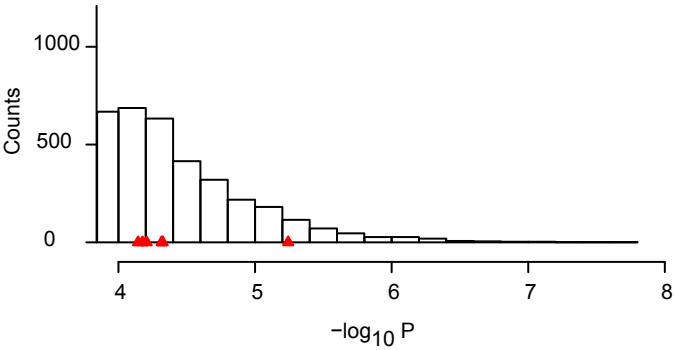

Supplement: Additional file 9 — Figure S6. Distribution of -log10 P values for ceQTLs in human noncoding regions. Noncoding ceQTLs closest to known lincRNAs and recently discovered unconventional genes are indicated by red arrows and tend to be among the lower -log10 P values. [file 1471-2164-12-562-S9.PDF]

FIGURE S7

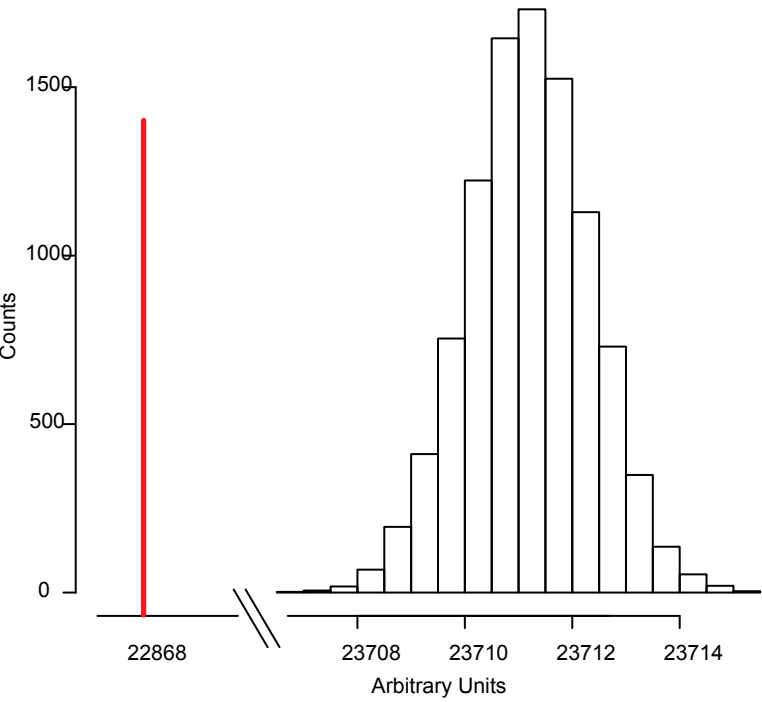

Supplement: Additional file 10 — Figure S7. Comparison between human RH and SymAtlas. Distribution of Frobenius norm values for distance between human RH and SymAtlas data using permuted expression values. Observed human RH-SymAtlas distance shown in red. Units are arbitrary. [file 1471-2164-12-562-S10.PDF]

FIGURE S8

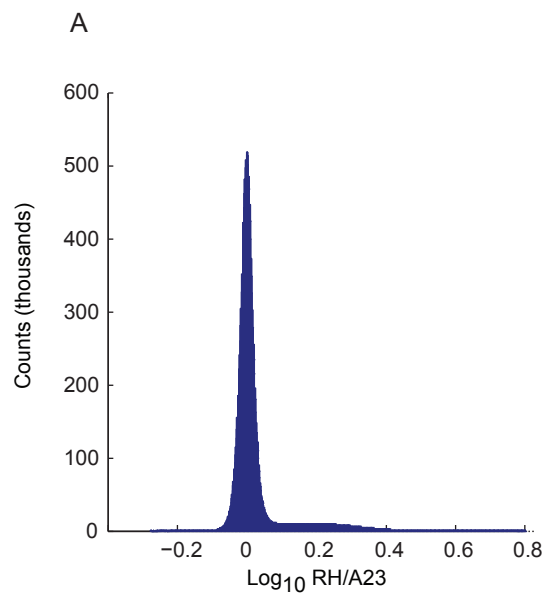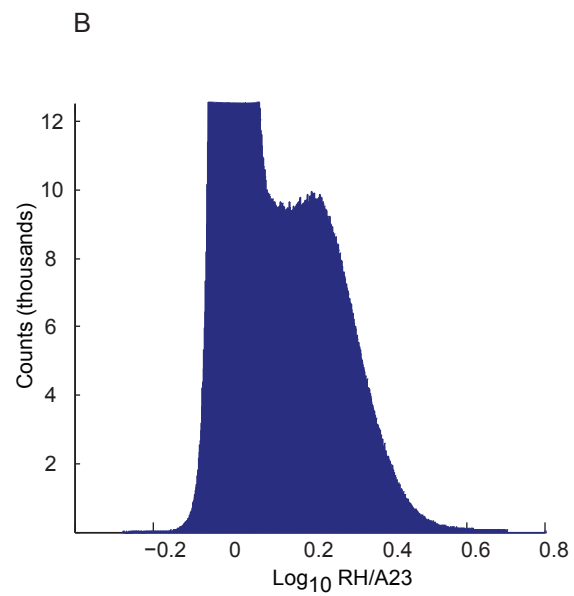

Supplement: Additional file 11 — Figure S8. Log10 (RH/A23) aCGH intensity data is bimodal. (A) Histogram of aCGH intensity for all RH clones. The large mode represents equivalent copy number between RH clones and hamster A23 control genomes, while the smaller mode to the right indicates markers with an extra copy in the RH clones. (B) Close up view of the second mode. [file 1471-2164-12-562-S11.PDF]

FIGURE S9

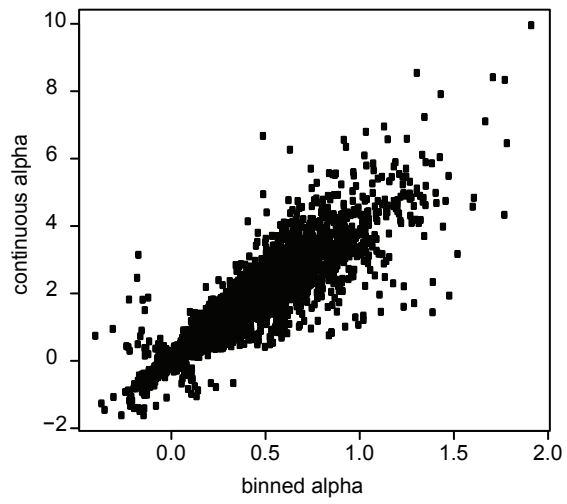

Supplement: Additional file 12 — Figure S9. Comparison of α from binned and continuous CGH data. CGH data was either binned into 0 or 1 extra copies or used as continuous values and used to calculate α. The correlation is 0.95, P < 2.2 × 10^-16^. [file 1471-2164-12-562-S12.PDF]
