# Supplementary material for: Effects of genome-wide copy number variation on expression in mammalian cells
Source: BMC Genomics. 2011 Nov 16;12:562. doi: 10.1186/1471-2164-12-562 (PMC3287593; doi:10.1186/1471-2164-12-562)
Supplement: Additional file 7 — Table S2. Common genes with negative cis α in mouse RH, human RH and TCGA. [file 1471-2164-12-562-S7.DOC]

**TABLE S2**

**Common genes with negative cis α in mouse RH, human RH and TCGA**

|  |  |  |
| --- | --- | --- |
| ACOXL | GZMM | NPPB |
| ADRA2C | HNF4G | PADI1 |
| AKAP4 | HOXD13 | PAQR6 |
| ALK | HPN | PAX5 |
| BMP10 | HS3ST3B1 | PLCL1 |
| CD96 | IL5 | PRODH2 |
| CHRNB3 | KCNN1 | RAG1 |
| CTSS | KERA | RELN |
| CYP7A1 | LHX3 | S100A9 |
| F9 | LPO | SCN4A |
| FABP2 | MAP3K10 | TNFRSF13B |
| FOXL2 | MIP | WNT11 |
| GALR2 | MLANA |  |
| GATA4 | MYL7 |  |
| GPR45 | NEU2 |  |
|  |  |  |
|  |  |  |
|  |  |  |
|  |  |  |
|  |  |  |
|  |  |  |
|  |  |  |
|  |  |  |
|  |  |  |
|  |  |  |
|  |  |  |
|  |  |  |
